# Modularity of the Human Musculoskeletal System: The Correlation between Functional Structures by Computer Tools Analysis

**DOI:** 10.3390/life12081186

**Published:** 2022-08-03

**Authors:** Daniele Della Posta, Jacopo Junio Valerio Branca, Giulia Guarnieri, Cristiana Veltro, Alessandra Pacini, Ferdinando Paternostro

**Affiliations:** Department of Experimental and Clinical Medicine, Anatomy and Histology Section, University of Firenze, L.go Brambilla 3, 50134 Firenze, Italy; osteodan@gmail.com (D.D.P.); jacopojuniovalerio.branca@unifi.it (J.J.V.B.); giulia.guarnieri@unifi.it (G.G.); fisioveltro@gmail.com (C.V.); alessandra.pacini@unifi.it (A.P.)

**Keywords:** algorithms, modularity, biological models, human body, anatomical structure, musculoskeletal system, locomotor apparatus

## Abstract

Introduction: For many years, anatomical studies have been conducted with a shattered view of the body. Although the study of the different apparatuses provides a systemic view of the human body, the reconstruction of the complex network of anatomical structures is crucial for the understanding of structural and functional integration. Aim: We used network analysis to investigate the connection between the whole-body osteo-myofascial structures of the human musculoskeletal system. Materials and Methods: The musculoskeletal network was performed using the aNETomy^®^ anatomical network with the implementation of the open-source software Cytoscape for data entry. Results: The initial graph was applied with a network consisting of 2298 body parts (nodes) and 7294 links, representing the musculoskeletal system. Considering the same weighted and unweighted osteo-myofascial network, a different distribution was obtained, suggesting both a topological organization and functional behavior of the network structure. Conclusions: Overall, we provide a deeply detailed anatomical network map of the whole-body musculoskeletal system that can be a useful tool for the comprehensive understanding of every single structure within the complex morphological organization, which could be of particular interest in the study of rehabilitation of movement dysfunctions.

## 1. Introduction

### 1.1. Biological Modularity

The modularity feature has a crucial role in biology, helping a system to ‘save its job’ and, in the meantime, allowing further evolution. The modularity’s advantage is commonly employed in engineering, leading to encouraging its usage as a model for evolutionary design [1]. However, biological systems are not designed by evolution but are shaped by it. Indeed, modularity is assumed to exist a priori and to be the best functional solution [2] even though the majority of the biological system function could be better performed without a modular approach. Many hypotheses have been suggested to explain how modularity emerges and by which conditions. Some theories argue that the selection criterion is not essential for modularity [3], while others speculation have explained the modularity arising both by a direct or indirect fitness benefit such as increased evolvability [4], facilitated horizontal gene transfer [5], or by the increased robustness [6,7]. However, most biologists agree on the existence of modularity, describing it in different ways [8]. A systems biologist might set modules from the point of view of the graph theory, describing them as groups of nodes that are strongly interconnected for chemical and physical reasons [9], a geneticist might consider a set of co-expressed or co-regulated genes as a module [10,11], and an evolutionary biologist might look for conserved sequences or structures [12].

On the other hand, modularity is an organizational property of biological systems that can be found at different levels. Individual elements (nucleotides, proteins, anatomical portions, etc.) can constitute a module, and different types of connections (both dynamic and static) can be used to determine it [13]. Thus, each biological component of an organism, although more or less heterogeneous and strongly interplaying within themselves, is split into groups that react relatively independently with each other, even though they belong to the same system.

However, as already mentioned, the modules are not independent and therefore can be studied from a co-evolutionary point of view. For example, the modules are generally considered as distinct parts that interact with each other during embryo morphogenesis, thus referred to as developmental or evolutionary modules. Indeed, once the interactions in adult individuals are conformed, modules are assumed to be made of cohesive parts, even belonging to different developmental modules, that together perform a physiological function or a relevant behavior and are therefore called functional modules. Thus, a module, whether variational, functional, or evolutionary, is a part of an organism that is integrated into a certain type of process (natural variation, physiological function, developmental process, etc.) and is relatively autonomous from the others.

### 1.2. The Modular Anatomical Organization of the Musculoskeletal System

The human body is a complex structure that responds to construction standard that is defined as bio-tensegrity. Tensegrity is an engineering concept that has also become widespread in biology for the description of cellular structure and biological tissues. The bio-tensegrity structures are self-organized and can react to external stimuli systemically by favoring the distribution of loads through the maintenance of constant tension of the bonds between the individual components, thus allowing an oscillation capable of generating a discontinuous compression [14]. Tensegrity is, therefore, a concept of mechanical complexity that satisfy the idea of structures able to respond in a systemic way to environmental stresses and are provided with particular robustness due to their modular design [15]. However, the whole human body, which has been described for years as a bio-tensegrity structure, and therefore capable of its self-regulation and functional autonomy, not entirely subordinate to the neural [16] hierarchy activity, is subdivided into modules mainly for functional aspects.

The human biomechanical network is divided into modules according to the criteria of centrality, bridgeness, and overlap and can be useful for identifying functional aspects, as reported by Csermely [17]. Csermely described active centers as nodes that coordinate the development and evolutionary tendencies of a network [17]. Indeed, the active centers are nodes that can connect two topological modules (bridges) or can represent specialized elements useful for restoring alteration of the topology and for transferring information in a network. In the latter case, the overlapping of modules could play a key role in the variability of functional activities, which requires disconnection and synchronization of different modules and their reorganization respectively during and after the activities. In this sense, border nodes (module overlap measure) and bridge nodes (module bridgeless measure) play an important role that guarantees both structural and functional continuity to the entire biomechanical network. Their role will be central in maintaining a regular morphological communication activity, which represents one of the peculiarities of the functional adaptability of biological structures. Furthermore, from a clinical point of view, the topological distribution of the nodes could be a valid predictor of the state of health of the patient’s musculoskeletal system [18].

It is worth mentioning that the first studies carried out on anatomical structure date back to 1785 with the morphological study. Indeed, J.W. Goethe [19] firstly coined this name to indicate the study of forms in comparative anatomy. In addition to the description of the variational and evolutionary modules, morphology has also been used to topographically divide anatomical parts into regions that comply with an isomorphism concept, as it happens for the thorax, describing the rigid “defenses” protecting vital organs or a functionalist notion as it happens for the limbs, articulated appendages of the body structure necessary, but not only, for walking and manipulation. Nowadays, the body’s areas, such as the shoulder or upper limb, are described by the aspects exclusively linked to their articular functional component. Unfortunately, the current topographical subdivision of the morphology of the structure corresponds to a limited and reductive point of view about the morphology concept. Morphology is a broader concept that encompasses a functional notion specific to the structure that may not be subordinated to functional specificity. The functionality of the limbs cannot be isolated from the thorax or the abdomen [20], and consequently, the current morphological definition of an upper or lower limb is incorrect. Indeed, the functional complexity hides an enormous structural intricacy that requires a more adequate morphological study, which is now possible thanks to the graphic and mathematical tools of network analysis.

### 1.3. Study on the Modularity of the Human Anatomical Biomechanical Network

To better investigate the morphological modularity from an anatomical point of view, based on the knowledge acquired through anatomical dissections, we decided to use an anatomical network model of the locomotor system to study the distribution of the modules through a quantitative analysis of the topography, highlighted by a graphic representation. The use of the network allowed us to observe the set of relationships between the anatomical parts of a system and to navigate within it as a map. Moreover, the geometric arrangement of the relationships between the parts allows a mathematical analysis of the graph characterizing the anatomical network, with the possibility of identifying hierarchies, clusters, and, therefore, anatomical modules according to the topology of the map.

A module of a network corresponds to a set of nodes, which have a great influence on each other.

Previous studies focused on the musculoskeletal modularity of portions of the body, such as the head and limbs, have already been carried out by several authors [21,22,23]. On the other hand, few studies have been devoted to the entire network of human musculoskeletal structure [24,25,26,27].

Therefore, the first aim of this study is to underline the modules of the whole musculoskeletal system according to topological features of the anatomical network of the musculoskeletal structure or the biomechanical network.

## 2. Materials and Methods

Generally, the modules of a network are identified independently from several attributes that characterize the nodes’ and the links’ network, but it is also possible to modularize the network without any specific attribute and therefore using an unweighted network [28]. The anatomical network used for the present study is the aNETomy^®^ (anatomical network project; EUIPO (European Union Intellectual Property Office) trademark number 012027447) and has already been used in previous work [http://anetomy.it/ (accessed on 11 March 2021)] [24,25,26,27,29]. This model is updated and includes the nomenclature of all anatomical parts mentioned in the skeletal, articular, and muscular systems according to the Second Edition of the International Anatomical Terminology, FIPAT (The Federative International Programme For Anatomical Terminology) [30]. The FIPAT encompasses the international standard on human anatomical terminology that firstly goes back to 1895 [31] and was then developed by the Federative Committee on Anatomical Terminology (FCAT) and the International Federation of Associations of Anatomists (IFAA), released in 1998 [32], continuously aimed to correct and extend the anatomical nomenclature in different areas such as neuroanatomy [33] and locomotor system [34]. The design of this anatomical network model is certainly the one that best conveys the concept of structural tensegrity. Generally, the network provides a simple model of a more complex system and can be useful in order to study the actions between the interacting parts, thus understanding the hierarchical complexity of the studied system. The anatomical network provided in this study consists of 2298 nodes representing the individual anatomical parts, assimilated to the rigid elements of the tensegrity structure, connected through 7294 links (i.e., the contact points between two anatomical parts and assimilated to the tensile elements of the tensegrity structure) [24]. The result is a system in which each anatomical part is a node that is joined to other anatomical parts by links, which are the physical contact points necessary for the functional dynamics. Therefore, functional subsystems, such as osteo-tendinous or osteo-ligamentous insertions, as well as articular systems, are represented by at least two nodes and a link.

Once aNETomy^®^ is loaded on Cytoscape (version 3.8.2; National Institute of General Medical Sciences (NIGMS), Bethesda, MD, USA) (an open-source software dedicated to the analysis and graphical visualization of networks mainly used in the biological field) [35], it is necessary to proceed with subsequent steps for the modularization of the network. First, the network is analyzed with the network analyzer [36] plug-in, which provides Cytoscape users an algorithm to calculate the topological quantities of nodes and links, whereupon we use the ModuLand [29,37] plug-in, also available from Cytoscape, for determining extensively overlapping network modules. ModuLand consists of several modularization methods and has already been widely used in different areas of research on networks [37,38]. Moreover, the tool assigns module cores, thus predicting the function of the whole module and determining key nodes bridging two or multiple modules [37].

**Figure 1:**
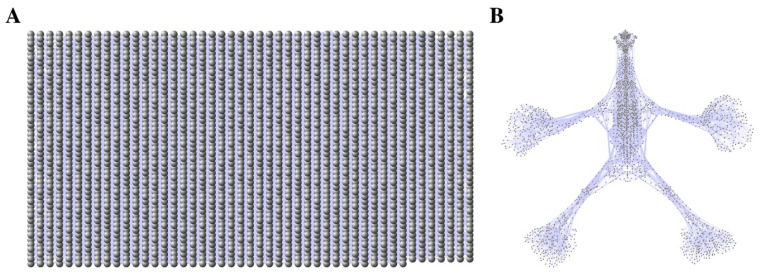
Anatomical network aNETomy^®^. The grid containing the 2298 nodes and the 7294 links (panel (**A**)) turns into an anthropomorphic network (panel (**B**)) after the Cytoscape force-directed layout application.

The anatomical network aNETomy^®^, with all its 2298 nodes and 7294 links, is shown in Figure 1. The initial grid on the left, thanks to a manually adjusted force-directed layout, evolves into an anthropomorphic image divided into two symmetrical portions, made up of two peripheral and lateral appendages and a central axial connection zone. At this level, the network has no attributes and is therefore not weighted. Moreover, it is an indirect network as the links do not own any directional vector.

Of all the quantities detected by the network analyzer algorithm, the one we use for the modular partition procedure is Edgebetwenness (Eb). The Eb is a topological quantity [39] and corresponds to the number of shortest paths through the links of a network. The higher the coefficient, the more a link is an important connector for the communication function between the nodes in a network. Indeed, each link is like a bridge, and if it owns a high Eb coefficient, it connects the central nodes of communities that constitute two portions of a network. Thus, its removal can affect the communication between many pairs of nodes in a network, leading to its global functioning alteration.

**Figure 2:**
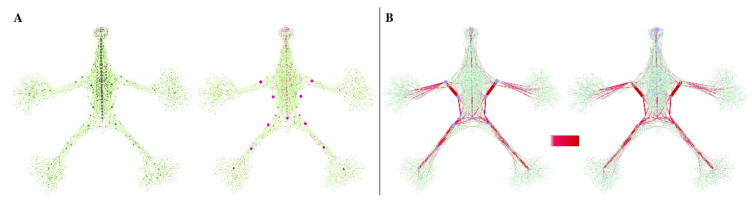
The Edgebetweenness (Eb) distribution in the network. (**A**) The pictures show the distribution of the counted nodes related to the Degree centrality and Betweenness centrality. The bigger black dots indicate the most relevant ones. On the left, it is shown the nodes with more links distributed in the axial region of the network. On the right, it is displayed the nodes with a more intermediation role that are located in the appendicular regions and crossing the central zone. (**B**) Following the chromatic gradient in the center, the links with a higher Edgebetweenness (Eb) can be recognized. On the left, the bigger dots have a higher degree, and on the right, the higher betweenness. As shown, both nodes correspond to the links with a higher Eb.

**Figure 3:**
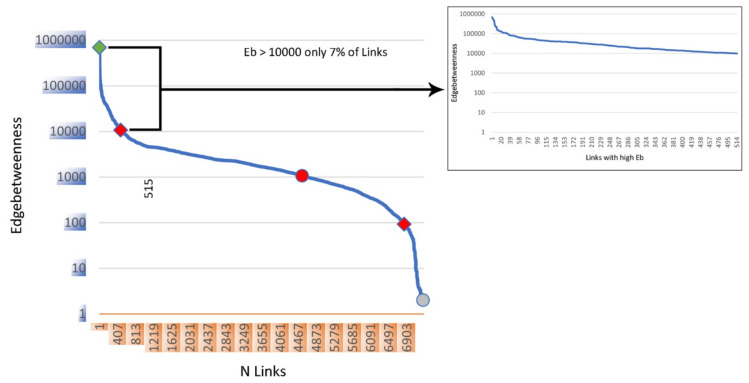
The Edgebetweenness (Eb) coefficient distribution. The graph displays the Eb distribution curve on the 7294 links of the anatomical network. It shows how the Eb coefficient, from 690,663 to 100,000, is distributed on a little part of the links’ network. This trend continues from 100,000 to 10,000, including 515 links that resemble 7% of the anatomical network. The majority of the links own an Eb coefficient under the 10,000 limits with an almost linear distribution until 100, followed by an abrupt fall of the Eb index for the remaining links.

**Figure 4:**
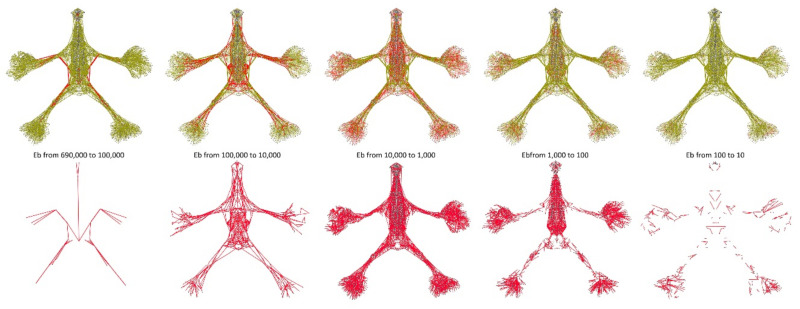
The Eb distribution following the link count (highlighted in red) within the anatomical network aNETomy^®^. In the upper panel is shown the whole network, while in the lower one, the links for each fascia are shown, respectively. The first two images on the left side of both upper and lower panels represent the directors that, with a clear geometrical organization, steer the shortest paths between the anatomical network components. On the other hand, in the middle, is evident the massive presence of the links with the Eb ranging in the third fascia (fascia 3, 10,000–1000). Finally, the figure shows how the links and the nodes of the network, in the last two fasciae, tend to be geometrically less organized and more reduced.

The distribution of Eb in our anatomical network is shown in Figure 2, Figure 3 and Figure 4.

**Figure 5:**
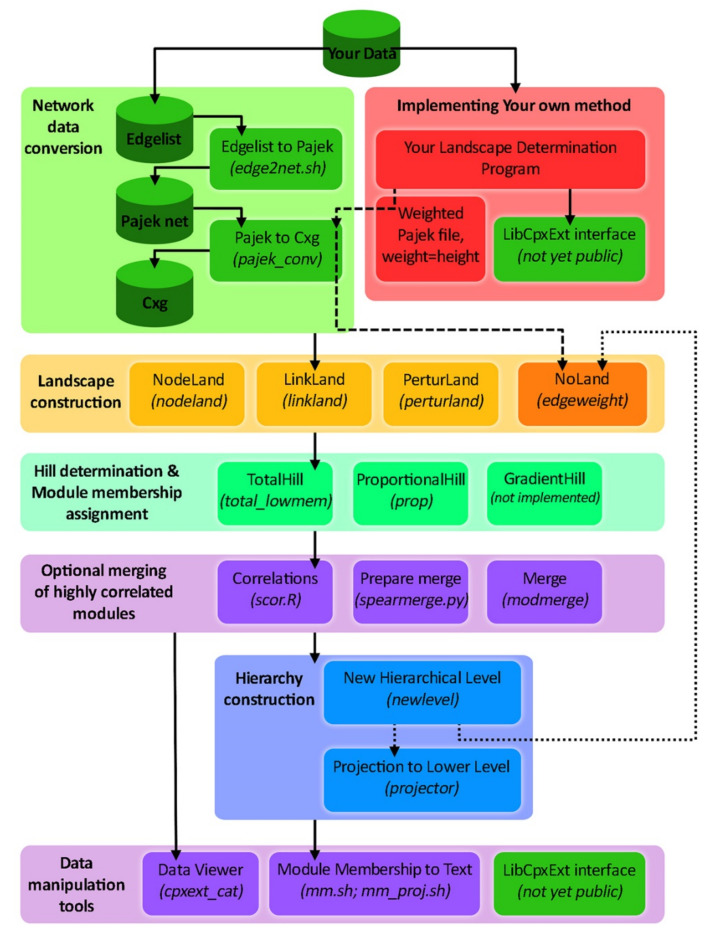
The ModuLand algorithm package and its different steps. In the present picture, the cylinders stand for the option data of the archiving, whereas the squares stand for the different operations. The square legend refers to the operation name, while the name between brackets refers to the program executable as it is in the ModuLand program [37,38]. Reprinted with permission from © 2010 Kovács et al. [38].

The analysis with the Eb attribution allows the use of the ModuLand algorithm, which is called LinkLand [39]. This algorithm works by calculating the different roles of each link in the network, activating four distinct phases (Figure 5).

**Figure 6:**
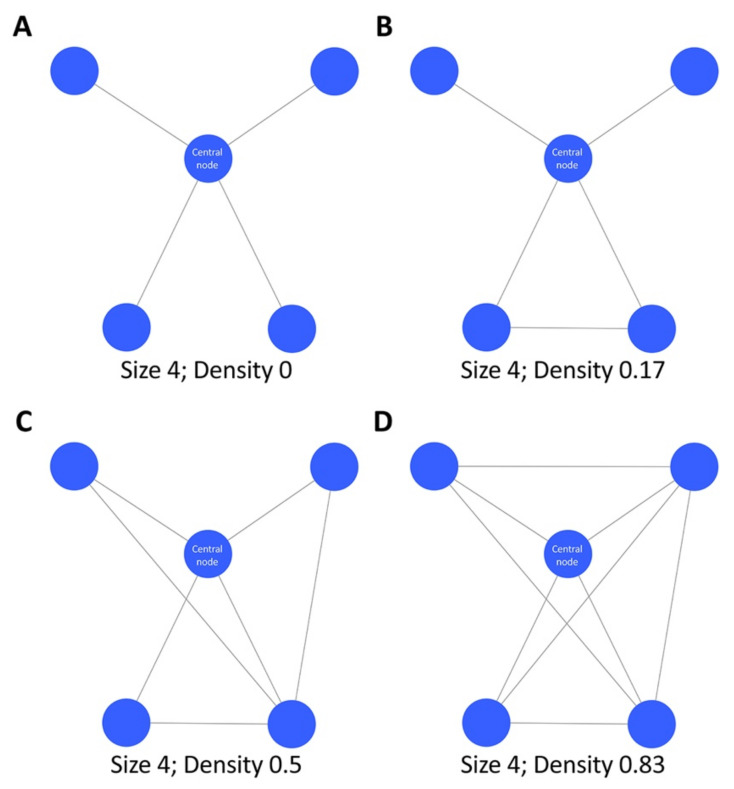
Density of a network. The four networks with the same size (4) present different densities (panel (**A**), density 0; panel (**B**), density 0.17; panel (**C**), density 0.5; panel (**D**), density 0.83).

The first phase includes the assignment of the influence zones characterized by density, a parameter showing the level of connectivity of the nodes in a network. The density of a network is defined as the ratio of actual links to potential links, and the higher its coefficient increases, the more clustered the included nodes are (Figure 6).

The LinkLand algorithm also considers the count of the links and, therefore, the density is defined by the ratio of the link weights sum ($w_{i,j}$) belonging to the initial influence zone to the number of nodes $\left(i,j\right)$, included in the influence zone |*A*|.

$$d=\frac{\sum_{\left(i,j\right)\in A}w_{i,j}}{\left|A\right|}$$

The origin point of each influence zone is given by a link of the total network with its two end nodes (*k*, *l*). Therefore, the initial density is the half weight of the starting link. The influence of the origin point extends involving the neighboring nodes and the links that connect them to the real influence zone until the connection strength of the influence function of the neighboring nodes to the realized influence zone is at least equal to the density of the influence zone.

The algorithm continues its iterative cycle by determining the influence function belonging to the next link in the original network ($f_{s}\left(i,j\right)=w_{i,j}w_{k,l},\;if\;\left(i,j\right)\in A$) if s equals or differs from 0.

If there are no further neighboring nodes with equal or greater connection strength of the influence function than the density of the influence zone, the influence function of the initial link is ready, and the density is recalculated.

$$d^{\prime }=\frac{\sum_{\left(i,j\right)\in A\cup \left\{k\right\}}w_{i,j}}{\left|A\right|+1}$$

The second phase provides the numerical series of values characterizing the influence coefficient of the links and nodes belonging to each module of the network that are necessary for the graphical representation of the overlapping modules distribution typical of the method, and that is developed in the third phase.

The nodes with reciprocal influence are detected considering the centrality coefficient of each link resulting in the sum of all the influence functions of each link in the network.

$$c\left(j,k\right)=\Sigma_{i}f_{i}\left(j,k\right)\geq 0$$

Once the link centrality coefficient has been calculated, the node centrality coefficient may be determined.

$$c\left(i\right)=\Sigma_{j}c\left(i,j\right)$$

In the third step, the plug-in develops a graphical representation in which the algorithm distributes the nodes of the network according to their hierarchies, representing it as a landscape with 3D graphics.

**Figure 7:**
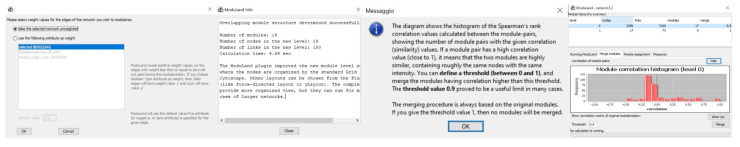
ModuLand algorithm function. The figures represent some of the analysis procedures of ModuLand, including, from the left side to the right one: the selection of the count link value; the analysis synthesis; the suggestions for the modules merge with 0.9 thresholds; the modules correction histogram.

Finally, in the fourth step, the plug-in develops a new network derived from the original one. A meta-network, called the next hierarchical level, consists of the “core” nodes (i.e., those with the greatest influence function) from which the name of each module derives. The procedure involves an important step which is the choice of the correlation coefficient that allows the identification of the correlation between pairs of modules. The coefficient is between 0 and 1, where 1 indicates that a pair of modules are strongly correlated, while 0 indicates no correlation. The “merge” function of the ModuLand algorithm (Figure 7) associates modules that have a coefficient above the defined threshold. 

Therefore, once we had chosen the value of the links to be used for the LinkLand analysis on the anatomical network, we started the algorithm by setting the correlation coefficient to 0.85. Thus, all four steps have been processed in a few seconds, restoring the original level 0 network divided into modules and the new level 1 network (Figure 7).

## 3. Results

**Figure 8:**
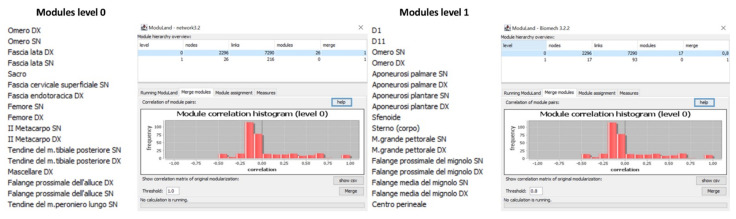
The unweighted network analysis. Modules names and correlation histogram of the unweighted network (network A) at 0 and 1 levels.

**Figure 9:**
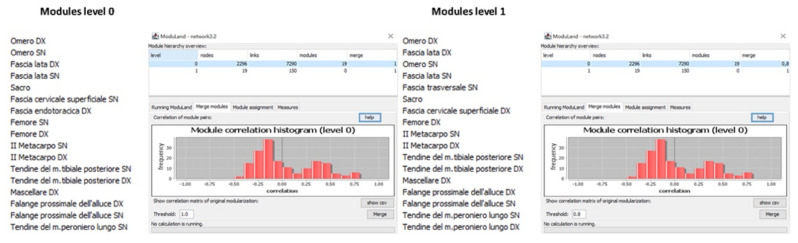
The unweighted network analysis. Modules names and correlation histogram of the weighted network (network B) at 0 and 1 levels.

The study was carried out on the same unweighted (next referred to as network A) and weighted (next referred to as network B) anatomical osteo-myofascial network. Initial network analysis calculated the presence at level 1 of 26 modules and 216 correlation links for network A (Figure 8) and 19 modules and 150 correlation links for network B (Figure 9). Then, the modularity coefficient was applied, setting the threshold at 0.8 for both networks. Thus, in network A the number of modules was reduced to 17 and the number of correlation links to 93, while in network B, the number of modules did not change since there were no correlations between modules above the threshold.

**Figure 10:**
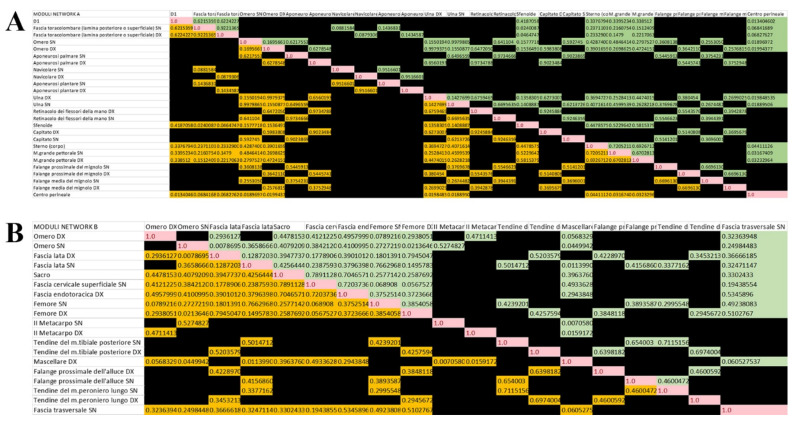
The correlation links values. The figure shows the matrix correlation of the network (**A**) (unweighted, upper panel) and (**B**) (weighted, lower panel) modules. The negative values are obscured.

In subgraph A, the majority of the 261 correlation links had a negative value indicating a lack of correlation between the two modules. For this reason, only positive indices are shown in the matrix, as reported in Figure 10. In addition, the algorithm also calculated a quantity called moduland_weight_lev_1, concerning the weight of each link in the network at level 1. By removing all the coefficients below the 0 value of the quantity moduland_weight_lev_1, the number of correlation links of subgraph A became 50.

However, also the negative correlation links in the correlation matrix of the network B modules were obscured. Moreover, by also removing the negative coefficients of the moduland_weight_lev_1 of the 150 correlation links, 91 remained.

**Figure 11:**
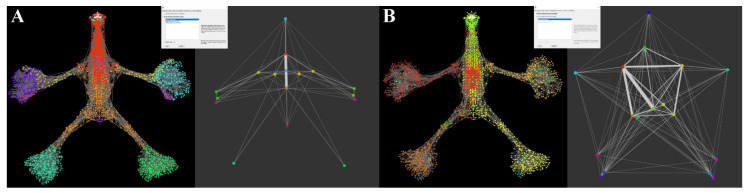
Modules distribution of the anatomical network aNETomy^®^. (**A**) On the left side, a network composed of 17 modules corresponding to the biomechanical network analyzed by ModuLand, without specific values. The corresponding geometrical picture on the right side corresponds to level 1 output developed from the fourth phase of the LinkLand algorithm. (**B**) It is shown a network formed by 16 modules corresponding to modules of the biomechanical network analyzed by ModuLand, referring to the Eb centrality. On the right side, level 1 contains the core nodes of each module. In both panels are also highlighted the links connecting the modules. The different thickness is due to the centrality values. The greater the thickness, the greater the degree of influence between two modules.

Now, if we look at the subgraphs of level 1 in Figure 11, it is clear the different distribution of the modules and the different forms of the network. Furthermore, in subgraph A, the symmetrical distribution of both nodes and links is clear. Indeed, the structure is given by five median axial nodes, two periaxial nodes, four for each upper limb, and one for each lower limb of graph A, which completely disappear in subgraph B, where the nodes also present an asymmetrical distribution together with the correlation links. 

Such a difference in the distribution between the two graphs suggests that the size Eb, although related to the topological organization of the network structure, may also represent a functional behavior of the structure itself. Indeed, the relations of a functional type between different areas of the body were already known, for example, in the neurophysiological field, as it happens, for the synchronous activity of the hands and feet or between the two lower limbs [40,41,42].

## 4. Discussion

The term modularity has been recently widely diffused in different research fields, including human anatomy. Even if each module of a specific system has an intrinsic function, this can be linked and overlapped with the other ones and generate new networks-modules, working together in an efficient way with different roles. For example, in evolutionary biology, modularity is linked to the evolutionary possibilities of an organism. Indeed, the more independent the modules are from each other, the more they will be able to respond independently to the selection. The morphological component of an organism is therefore shared into “variational modules” because a set of features can independently change in comparison to the evolution of other traits.

Taking into account the anatomical-functional partition that considers the existence of different systems (nervous, vascular, endocrine, muscular, etc.) [43], due to the different histological characteristics of the constituent cells, we arrived at a further partitioning by specific functional criteria. Indeed, for example, the muscular system is further subdivided into modules called muscle groups that perform specific movement activities, such as pronators and supinators of the forearm [43]. Over the years, this concept has been further extended to the skeletal and arthrodial structures involved in the execution of a gesture through patterns of functional myofascial synergy [44]. However, although the determined myofascial modules extended the complexity of the anatomical modularity concept within the body movement, they add little to the modularity of structure. Thus, since the present project is carried out on models of anatomical networks, it might allow overcoming the limitations of the current topographic division, allowing the development of more realistic biomechanical evaluation procedures of the movement.

Indeed, in our study, being faced with anatomical modules that are physically connected by tendinous or ligamentous junctions, a strong link between neighboring modules seems clearer, but evaluating the magnitudes of Eb and moduland_weight_lev_1, it is shown that there are favorable paths of communication between groups or single nodes. Therefore, it can be hypothesized that the connection between two anatomical structures should not be seen only by a direct biomechanical–structural relationship but by the action of influence that the parts of a module act on the neighboring ones following the mentioned mechanisms of modular density. Such a hypothesis refers to the concept of morphological communication, where the osteo-myofascial system transmits information of a mechanical type within itself to activate the necessary responses to the movement functions. This concept, only recently introduced in the biomechanical field, supports a new decentralized and, above all, non-neuronal vision of the human movement. Studies carried out by Rieffel and Valero-Cuevas, confirm the idea that morphology, either of a single anatomical part or an anatomical module, represents an entity with its computational capacity (Embodied Anatomical Computation) for the transmission of forces and movement in the space of large modular systems [45]. Valero-Cuevas and colleagues demonstrated, by computer simulations on cadaveric experiments performed on hand fingers, that the tendon network achieves logic computation to preferentially change torque production capabilities of phalanxes. Indeed, by systematically changing the proportion of tension to the tendons versus different muscles, the authors found that the distribution of input tensions in the tendon network itself regulates how tensions propagate to the finger joints, thus acting as a switching function of a logic gate [16]. Such a concept can be referred to as the term “morphosis”, which requires plasticity of morphology that derives not only from the elasticity of the tissues but also from their topological organization, thus allowing a new morphological organization together with a movement reprogramming according to environmental needs [46].

The morphological action is closely related to passive biomechanical dynamics [47] and has more of transmitting information rather than promoting dynamic stability to the individual module or the whole body. We know that, according to the Maupertuis principle of minimum action [48], the synergistic coordination of the musculoskeletal system develops on preferential communication paths between individual parts or anatomical modules that, in case of functional overload, will be supported by auxiliary or compensatory paths. However, the clinical experience that has been conducted so far teaches us that these pathways are hierarchically identifiable through the Eb coefficient of the individual nodes and/or the correlation coefficient between anatomical modules.

Lastly, even if the outcomes highlight a different organization of modularity, the limitation of our preliminary study is mainly linked to being just an anatomical model. In this regard, for example, no gender or anatomical variability was analyzed. Indeed, the future perspective aims to analyze different specific body parts (such as the neck, arms, legs, and so on) together with more anatomical and model updates.

## 5. Conclusions

In conclusion, the present study identifies and suggests a map of correlations based on the influence forces of the anatomical modules, which could be of particular interest in the rehabilitation of movement dysfunctions. Such a map might be useful to look for dysfunctional modules that may alter the local or general biomechanical function of the musculoskeletal system, even if far from the area of interest. Furthermore, it could be a valuable tool, especially for the clinician/therapist who practices movement rehabilitation according to the criteria of globality. Indeed, the knowledge of the anatomical features and the interplay between different parts and tissue is fundamental for different professionals such as phyosiotherapists and osteopaths, as well as for the valuations and therapies application of the locomotor apparatus and dysfunctions of the movement framed in a holistic a systemic point of view. However, a deep knowledge not only of anatomical aspects but also of informatics algorithms is needed.
